# *Chlorella vulgaris* Modulates Genes and Muscle-Specific microRNAs Expression to Promote Myoblast Differentiation in Culture

**DOI:** 10.1155/2019/8394648

**Published:** 2019-07-21

**Authors:** Nurhazirah Zainul Azlan, Yasmin Anum Mohd Yusof, Ekram Alias, Suzana Makpol

**Affiliations:** ^1^Department of Biochemistry, Faculty of Medicine, Level 17, Preclinical Building, Universiti Kebangsaan Malaysia Medical Centre, Jalan Yaacob Latif, Bandar Tun Razak, Cheras, 56000 Kuala Lumpur, Malaysia; ^2^Department of Basic Medical Sciences for Nursing, Kulliyyah of Nursing, International Islamic University Malaysia, P.O. Box 141, 25710 Kuantan, Pahang, Malaysia

## Abstract

**Background:**

Loss of skeletal muscle mass, strength, and function due to gradual decline in the regeneration of skeletal muscle fibers was observed with advancing age. This condition is known as sarcopenia. Myogenic regulatory factors (MRFs) are essential in muscle regeneration as its activation leads to the differentiation of myoblasts to myofibers.* Chlorella vulgaris* is a coccoid green eukaryotic microalga that contains highly nutritious substances and has been reported for its pharmaceutical effects. The aim of this study was to determine the effect of* C. vulgaris *on the regulation of MRFs and myomiRs expression in young and senescent myoblasts during differentiation* in vitro*.

**Methods:**

Human skeletal muscle myoblast (HSMM) cells were cultured and serial passaging was carried out to obtain young and senescent cells. The cells were then treated with* C. vulgaris* followed by differentiation induction. The expression of* Pax7*,* MyoD1*,* Myf5*,* MEF2C, IGF1R, MYOG, TNNT1, PTEN, *and* MYH2 *genes and miR-133b, miR-206, and miR-486 was determined in untreated and* C. vulgaris-*treated myoblasts on Days 0, 1, 3, 5, and 7 of differentiation.

**Results:**

The expression of* Pax7*,* MyoD1*,* Myf5, MEF2C*,* IGF1R, MYOG*,* TNNT1,* and* PTEN* in control senescent myoblasts was significantly decreased on Day 0 of differentiation (p<0.05). Treatment with* C. vulgaris *upregulated* Pax7*,* Myf5, MEF2C*,* IGF1R, MYOG*, and* PTEN *in senescent myoblasts (p<0.05) and upregulated* Pax7* and* MYOG* in young myoblasts (p<0.05). The expression of* MyoD1 *and* Myf5 *in young myoblasts however was significantly decreased on Day 0 of differentiation (p<0.05). During differentiation, the expression of these genes was increased with* C. vulgaris *treatment. Further analysis on myomiRs expression showed that miR-133b, miR-206, and miR-486 were significantly downregulated in senescent myoblasts on Day 0 of differentiation which was upregulated by* C. vulgaris *treatment (p<0.05). During differentiation, the expression of miR-133b and miR-206 was significantly increased with* C. vulgaris *treatment in both young and senescent myoblasts (p<0.05). However, no significant change was observed on the expression of miR-486 with* C. vulgaris *treatment.

**Conclusions:**

* C. vulgaris* demonstrated the modulatory effects on the expression of MRFs and myomiRs during proliferation and differentiation of myoblasts in culture. These findings may indicate the beneficial effect of* C. vulgaris *in muscle regeneration during ageing thus may prevent sarcopenia in the elderly.

## 1. Introduction

Muscle weakness and atrophy occur in ageing due to multifactorial degenerative processes. It is impacted by cellular ageing biology or primary ageing besides environmental and behavioral factors [1]. The term “sarcopenia” is derived from Greek words “sarx” and “penia”, which mean flesh poverty. It was first introduced by Rosenberg in 1988, in referring to the lean body mass loss in ageing [2, 3]. The diagnosis of sarcopenia can be based on the presence of both low muscle mass and low gait speed [3]. Sarcopenia is also defined as the age-related decline of muscle mass, function, and/or strength, with high prevalence in ageing [4]. It has been reported that muscle strength loss at 2.5% to 3.0% per year in women and 3.0% to 4.0% per year in men, while muscle mass loss at 0.64% to 0.70% per year in women and 0.80% to 0.98% per year in men, in people aged 75 years [5].

Myogenesis is the process of myoblast cells generation which occurs during skeletal muscle tissue synthesis [6]. The satellite cell is important as it is the source of myogenic cells required for myofiber growth and repair throughout life. Proliferation of satellite cell gives rise to satellite cell-derived myoblasts that can differentiate and form multinucleated myotubes [7]. The myogenic regulatory factors (MRFs) are the member of the basic helix-loop-helix family of transcription factors that control the differentiation of skeletal muscle cells during regeneration of muscle [8]. The MRFs consist of four muscle-specific proteins that include Myf5, MyoD, Myogenin, and Myogenic Regulatory Factor 4 (MRF4). These proteins act at multiple points in the muscle lineage to cooperatively establish the skeletal muscle phenotype. It regulates precursor cell proliferation and cell cycle arrest and activates sarcomeric and muscle-specific genes to facilitate differentiation and sarcomere assembly [9].

The satellite cells remain in quiescent state under normal condition. However, when muscle is damaged, the satellite cells are activated and reenter cell cycle phases to produce muscle progenitor cells that are able to regenerate new muscle fibers. In ageing, the satellite cells as well as the systemic and niche environment undergo changes that affect the regenerative functions of the muscle. Aged satellite cells lose their reversible quiescent state due to the upregulation of gene encoding for p16^INK4a^, a regulator protein of cellular senescence. Furthermore, the disruption of FGF2-Spryl1 signaling and delocalization of *β*1-integrin in old satellite cells leads to the disturbance of quiescence state and induction of p16^INK4a^ which provokes a switch of senescent-like state in becoming presenescent cells. This process will consequently impair the regeneration of skeletal muscle which includes activation, proliferation, and self-renewal of myoblasts [10–12].

MicroRNAs (miRNAs) are evolutionary conserved, small RNAs with about 17 to 22 nucleotides. It plays vital roles in gene regulatory networks by binding and repressing the activity of specific target mRNAs. MicroRNA has shown its potential as biomarkers in diseases such as cancer [13, 14]. miRNAs are also vital in the posttranscriptional control of gene expression [15, 16]. Several types of miRNAs are highly expressed in different types of tissue and cell, with most of them being tissue-specific. A previous study reported that mature miRNA expression is 20-fold higher than the mean expression of miRNA in other tissues indicating its specificity. Moreover, the expression of miRNA in tissue-enriched with mature miRNA is higher than in other tissues but less than 20-fold [17].

The miRNAs are divided into two categories: (1) miRNAs that are expressed specifically in muscle and not in other tissues known as myomiRs, and (2) miRNA that are expressed in nonmuscle tissue or broadly expressed in other cell types. The myomiRs are important in controlling skeletal muscle development and function as it may affect several biological pathways involved in myoblast proliferation, differentiation, and muscle regeneration [14, 18]. Subsets of myomiRs can be categorized as striated muscle-specific (miR-1, miR-133a, miR-133b, miR-206, miR-208a, miR-208b, and mir-499) or muscle-enriched (miR-486). Some of the myomiRs are expressed in skeletal muscle as well as in cardiac muscle. However, miR-133b and miR-206 are skeletal muscle-specific, and miR-208a is cardiac muscle-specific [16].

World Health Organization (2015) reported that there will be an increase in the number of older population with age between 70 to 80 years in 2050 [19]. And among the major problems in the elderly, sarcopenia is of importance. Taking a natural remedy such as microalgae may be beneficial as one of the approaches in the prevention and management of this condition. Microalgae are believed to be first consumed by human 2000 years ago when the Chinese used blue-green algae,* Nostoc* to survive during the scarcity of food [20]. Another alga,* Chlorella,* has been used in variety of biotechnology applications such as biodiesel production and biosorption of heavy metals [21–23].* Chlorella*, a genus of unicellular green algae, is the most popular photosynthetic microalgae being studied and investigated currently.

A Dutch researcher, Martinus Willem Beijerinck, discovered* C. vulgaris* 129 years ago and described it as coccoid green algal “balls” with a well-defined nucleus [23, 24]. The natural components found in* C. vulgaris* may be responsible for its pharmacological actions. Several studies reported* C. vulgaris* pharmacological effects, and this includes its antidiabetic [25, 26] and anticancer actions [27–29]. It also possesses highly nutritive contents such as carbohydrates, proteins, nucleic acids, chlorophylls, vitamins, and minerals [30] besides having *β*-carotene, lutein, chlorophyll-a, chlorophyll-b, ascorbic acid, tocopherol, riboflavin, and retinol [31, 32]. In this study, we aimed to determine the effects of* Chlorella vulgaris *on the expression of genes and myomiRs involved in muscle differentiation of young and senescent myoblast cells in an attempt to elucidate the mechanism involved during myoblasts differentiation. The finding of this study may provide current information regarding the properties of* C. vulgaris *that can be used for the prevention of sarcopenia.

## 2. Materials and Methods

### 2.1. Cell Culture

Human Skeletal Muscle Myoblasts (HSMM) from 20 years old female Caucasian donor were purchased from Lonza (Walkersville, MD, USA). The skeletal muscle myoblasts were cultured in Skeletal Muscle Basal Medium (SkBM) with fetal bovine serum (FBS), L-glutamine, human epidermal growth factor (hEGF), dexamethasone, and gentamicin/amphotericin-B as supplements to the media (Lonza, Walkersville, MD, USA). Cells were cultivated at 37°C with 5% CO_2_ atmosphere. The skeletal muscle myoblast cell then underwent serial passaging until it reached cellular senescence. The population doubling (PD) of the cell was calculated for each passage according to the formula ln (N/n)/ln 2 as N is the number of cells at harvest stage and n is the number of cells at seeding stage [33]. The starting PD for this research was PD 8. The skeletal muscle myoblast cells reached cellular senescence when the cells were unable to proliferate in culture, even with consecutive replenishment. Myoblast cells were considered young at PD 14 and senescent at PD 21.

### 2.2. Preparation of* Chlorella vulgaris *for Cell Treatment

Stock of* C. vulgaris *Beijerinck (strain 072) was obtained from the University of Malaya Algae Culture Collection (UMACC, Malaysia). The stock was grown in Bold's Basal Media (BBM) with a 12 h dark and 12 h light cycle. The algae were then harvested by centrifugation at 1000 rpm and dried by using freeze dryer. Later, the algae were mixed in distilled water at a concentration of 10% (w/v) and boiled at 100°C for 20 min by reflux method. The alga was centrifuged and lyophilized using a freeze dryer to obtain* C. vulgaris* in powder form [29, 30, 34]. Myoblast cells which were cultured in T25 culture flask were treated with* C. vulgaris* at concentrations of 10 and 100 *μ*g/ml [34]. After plating the myoblasts for 24 h and the cells reached 70 to 80% confluency, the culture medium was replaced with* C. vulgaris* containing medium and left for 24 h in a CO_2_ incubator at 37°C. The culture media were then replaced with differentiation medium and parameters were measured on Days 0, 1, 3, 5, and 7 of differentiation induction.

### 2.3. Immunofluorescence Staining of Myoblasts

Immunofluorescence staining on myoblasts in culture was performed using an antibody specific for desmin, at a dilution of 1:50 (D33; DAKO, Glostrup, Denmark). Skeletal myoblast cells were washed with phosphate-buffered saline (PBS) and fixed in 100% cold ethanol, followed by incubation in 1% fetal bovine serum (FBS). The skeletal myoblast cells were washed again with PBS and incubated consequently with anti-desmin monoclonal antibody (D33, DAKO, Glostrup, Denmark) and Alexa Flour 488 goat anti-mouse in dark environment (Life Technologies, Carlsbad, CA, USA) with PBS washing in between the two antibodies incubation. Cell nuclei were visualized by Hoechst 33342 (Life Technologies, Carlsbad, CA, USA). The desmin staining was viewed under EVOS FL Digital Inverted Fluorescence Microscope (Life Technologies, Carlsbad, CA, USA). The morphological changes of the myoblast cell were observed throughout the differentiation days.

### 2.4. Induction of Myogenic Differentiation

For induction of muscle cell differentiation, the proliferation medium SkBM was replaced with a differentiation medium; DMEM: F12 (Lonza, Walkersville, MD, USA) with supplementation of 2% horse serum (ATCC, Baltimore, USA). The differentiation medium was changed every two days until the desired day of differentiation for RNA extraction.

### 2.5. RNA Extraction

The extraction of RNA was carried out on Days 0, 1, 3, 5, and 7 of differentiation. Total RNA was isolated using TRI® reagent (Molecular Research Center Inc, Ohio, USA) on Days 0, 1, 3, 5, and 7 of differentiation. A total of 2 ml TRI reagent was added to 25 cm^2^ culture flask of seeding cells and left for 2 min prior to aliquot into microcentrifuge tube. Chloroform was added at 200 *μ*l and vortex before being left at room temperature for 15 min, followed by vortex at 12000 g and 4°C, for 15 min. The resultant clear layer of RNA was transferred into another new tube prior adding 500 *μ*l isopropanol and 5 *μ*l poly acryl carrier and left at room temperature for 5 min before centrifuged at 12000 g and 4°C for 8 min. The supernatant was removed and the RNA pellet was cleaned with 1 ml of 75% ethanol, followed by centrifuged at 7500 g and 4°C, for 5 min. Supernatant was further removed and RNA pellet was left to dry for about 20 min by directing the open tube to the laminar flow chamber. The dried pellet was later dissolved in 20 *μ*l RNase-free water (Life Technologies, New York, USA) and vortexed to be further kept at −80°C.

### 2.6. RNA Purity and Concentration

The purity of the extracted RNA was determined by Nanodrop 2000c Spectrophotometer (Thermo Scientific, USA). The extracted RNA with A260/280 value in a range of 1.8 to 2.0 was considered pure and used in gene and myomiRs expression analysis.

### 2.7. Primer Design

The primer of desired genes was designed from the National Center for Biotechnology Information (NCBI) gene bank. All of the primers used in gene expression analysis were synthesized by Integrated DNA Technologies, Inc. (Illinois, USA) except the primers for* Pax7*, which was synthesized from Bio Basic Inc. (Markham, ON, Canada) (Table 1). The target sequences for myomiRs expression analysis were synthesized by Life Technologies Corporation (Texas, USA) (Table 2).

### 2.8. Gene Expression Analysis

Gene expression analysis was carried out by using KAPA SYBR® FAST Bio-Rad iCycler® One-Step RT-qPCR Kit (Kapa Biosystems Pty. Ltd., Boston, Massachusetts, USA) according to manufacturer's instructions. The PCR mixture was prepared by mixing 0.3 *μ*l RT mix, 9 *μ*l master mix, 12.7 *μ*l RNase-free water, 1.0 *μ*l of 100 *μ*M forward primer, and 1.0 *μ*l of 100 *μ*M reverse primer of desired gene. A volume of 24 *μ*l PCR mixture was added to the desired well of 96-well PCR plate and 1 *μ*l of total RNA sample was also added. The 96-well plate was placed in CFX96 Touch™ Real-Time PCR Detection System (Biorad, California, USA). The protocol of one-step RT-qPCR was cDNA synthesis at 42° for 5 min, inactivation of reverse-transcriptase (RT) at 95°C for 4 min, followed by 40 cycles of 95°C for 3 sec and 53°C for 20 sec. The melting curve and data analysis were then generated at 95°C for a minute, 95°C for 30 sec, 55°C for a min, and 81 cycles of 55°C to 95°C for 10 sec. Primer specificity was then determined from the melting curve produced. All values of the threshold cycle (Ct value) obtained were normalized with the reference gene* GAPDH*. Relative expression value (REV) was calculated based on the relative quantitative method 2^-(ΔCt)^[35] by (1):

(1) $$\begin{array}{c} REV=2^{\left(The\;\;value\;\;of\;\;GAPDH\;\;Ct\text{-}The\;\;value\;\;of\;\;the\;\;target\;\;gene\;\;Ct\right).} \end{array}$$

### 2.9. MyomiRs Expression Analysis

The determination of miRNA expression was carried out by using TaqMan® Advanced miRNA Assays (Life Technologies Corporation, Texas, USA) based on the manufacturer's protocol. The poly (A) tailing reaction was prepared firstly by mixing 2 *μ*l of total RNA sample and poly (A) reaction mix in reaction tubes, consisting of 0.5 *μ*l 10X poly (A) buffer, 0.5 *μ*l ATP, 0.3 *μ*l poly (A) enzyme, and 1.7 *μ*l RNase-free water, which was then incubated in the thermal cycler for polyadenylation at 37°C for 45 min and stop reaction at 65°C for 10 min. Then, adaptor ligation reaction was performed by adding the ligation reaction mix which contains 3 *μ*l 5X DNA ligase buffer, 4.5 *μ*l 50% PEG 8000, 0.6 *μ*l 25X ligation adaptor, 1.5 *μ*l RNA ligase, and 0.4 *μ*l RNase-free water, into the reaction tubes containing poly (A) tailing reaction product and incubated for ligation at 16°C for 60 min. This is followed by RT reaction by adding the RT reaction mix, consisting of 6 *μ*l 5X RT buffer, 1.2 *μ*l dNTP mix, 1.5 *μ*l 20X universal RT primer, 3 *μ*l 10X RT enzyme mix, and 3.3 *μ*l RNase-free water, into the reaction tubes which contain the adaptor ligation reaction product and incubated for RT reaction at 42°C for 15 min and stop reaction at 85°C for 5 min. The miR-Amp reaction was then continued by mixing miR-Amp reaction mix containing 25 *μ*l 2X miR-Amp master mix, 2.5 *μ*l 20X miR-Amp primer mix, and 17.5 *μ*l RNase-free water and RT reaction product and incubated for enzyme activation at 95°C for 5 min, 14 cycles of denaturation at 95°C for 3 sec, and annealing/extension at 60°C for 30 sec and stop reaction at 99°C for 10 min. The miR-Amp reaction product was further subjected to RT-PCR reaction. A total of 5 *μ*l diluted cDNA template was mixed with 10 *μ*l Taqman® Fast Advanced Master Mix, 1 *μ*l Taqman® Advanced miRNA Assay, and 4 *μ*l RNase-free water and run for enzyme activation at 95°C for 20 sec and 40 cycles of denaturation at 95°C for 3 sec and annealing/extension at 60°C for 30 sec. Data of the threshold cycle (Ct value) acquired was normalized with the reference endogenous control hsa-miR-191-5p. The relative expression value (REV) was then calculated based on the relative quantitative method 2^-(ΔCt)^.

### 2.10. Statistical Analysis

Data obtained were expressed as mean ± SD and statistical analysis was carried out using SPSS software version 23. Data were analysed using one-way ANOVA followed by Tukey's post hoc test for multiple comparison. Value of p<0.05 was considered statistically significant.

## 3. Results

### 3.1. Immunofluorescence Staining of Myoblasts

**Figure 1:**
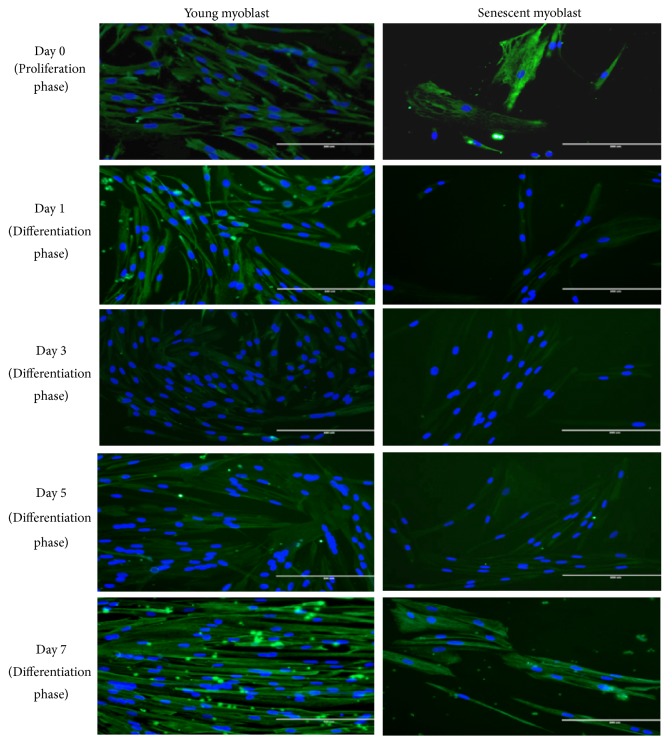
Effects of* C. vulgaris* treatment on desmin staining on Days 0, 1, 3, 5, and 7 of differentiation for young and senescent myoblast. The photomicrographs of desmin staining of* C. vulgaris*-treated myoblast cells further elucidated differentiation of myoblasts with the presence of multinucleated cells and more formation of myotubes on Day 7 as compared to Day 0 of differentiation. Myoblasts cells were stained green for desmin and blue for nuclei (Magnification: 200x).

Myoblast cells at young stage were spindle-shaped with less branches and underwent changes in morphology at senescent stage on Day 0 of differentiation. Senescent myoblast cells were larger and flatter with the presence of prominent intermediate filaments and single nuclei for each myoblast (Figure 1). Throughout the differentiation days,* C. vulgaris*-treated young and senescent myoblast cells were observed to differentiate, as notably indicated by the fusion of nuclei per myotube on Day 3 of differentiation and more formation of myotubes on Day 7 compared to Day 0 of differentiation. However, the differentiation of senescent myoblasts was observed to be less efficient as compared to young myoblasts for both the untreated and* C. vulgaris*-treated cells.

### 3.2. Gene Expression at Day 0 of Differentiation

**Figure 2:**
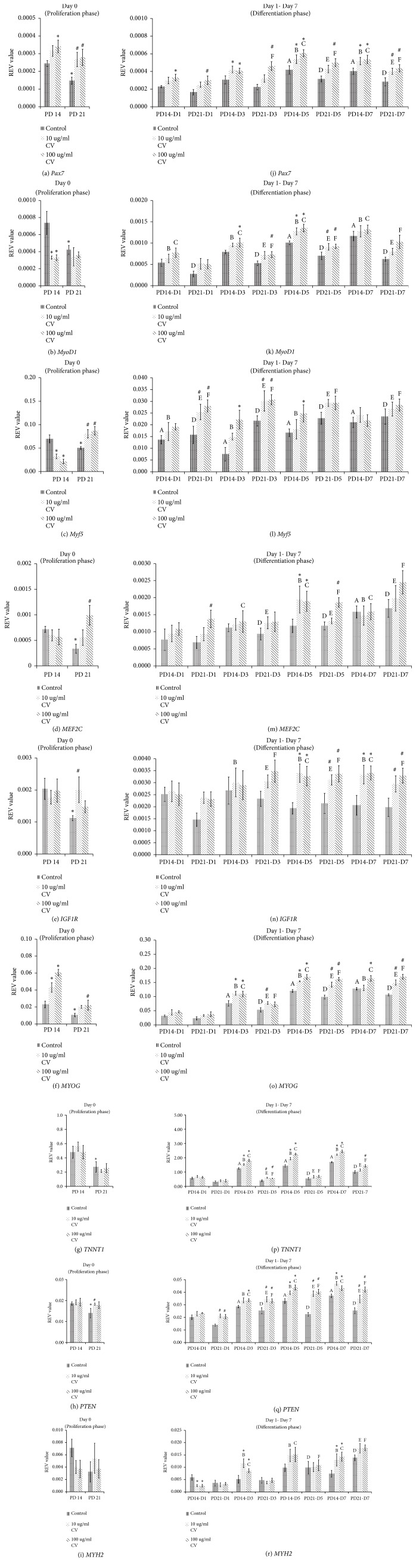
Effects of* C. vulgaris* treatment on the gene expression on Day 0 of differentiation (a-i) and Days 1, 3, 5, and 7 of differentiation (j-r). The expression of gene was determined in control and* C. vulgaris-*treated young and senescent myoblasts. The data are presented as the means ± SD, n = 3. *∗*p<0.05 significantly different compared to control young on respective day, ^#^p<0.05 significantly different compared to control senescent on respective day, ^A^p<0.05 significantly different compared to control young on Day 0, ^B^p<0.05 significantly different compared to young treated with 10 *μ*g/ml* C. vulgaris *on Day 0, ^C^p<0.05 significantly different compared to young treated with 100 *μ*g/ml* C. vulgaris *on Day 0, ^D^p<0.05 significantly different compared to control senescent on Day 0, ^E^p<0.05 significantly different compared to senescent treated with 10 *μ*g/ml* C. vulgaris *on Day 0, and ^F^p<0.05 significantly different compared to senescent treated with 100 *μ*g/ml* C. vulgaris *on Day 0, with a post hoc Tukey HSD test.

On Day 0 of differentiation,* Pax7, MyoD1*,* Myf5*,* MEF2C*,* IGF1R*,* MYOG*,* TNNT1,* and* PTEN* were significantly downregulated in control senescent myoblasts as compared to control young myoblasts (p<0.05) (Figures 2(a)–2(i)). However,* Pax7 *was significantly upregulated on Day 0 of differentiation with 100 *μ*g/ml* C. vulgaris *treatment in young myoblasts (p<0.05) (Figure 2(a)). A similar increase was observed in the expression of Pax7 (Figure 2(a)) and* Myf5 *(Figure 2(c)) in senescent myoblasts treated with 10 *μ*g/ml and 100 *μ*g/ml* C. vulgaris *(p<0.05). Decreased* MyoD1* (Figure 2(b)) and* Myf5* (Figure 2(c)) were observed in young myoblasts treated with both 10 *μ*g/ml and 100 *μ*g/ml* C. vulgaris* (p<0.05). Treatment with 100 *μ*g/ml* C. vulgaris *in senescent myoblasts significantly increased the expression of* MEF2C *(Figure 2(d)) and* MYOG *(Figure 2(f)) (p<0.05).* MYOG* was also significantly upregulated in young myoblasts with both 10 *μ*g/ml and 100 *μ*g/ml* C. vulgaris* treatment (p<0.05) (Figure 2(f)).* C. vulgaris *treatment at 10 *μ*g/ml in senescent myoblast significantly increased the expression of* IGF1R* (Figure 2(e)) and* PTEN* (Figure 2(h)) compared to control senescent myoblasts (p<0.05). The expression of* TNNT1 *and* MYH2 *however was not affected by* C. vulgaris *treatment in both young and senescent myoblasts (Figures 2(g)–2(i)).

### 3.3. Gene Expression on Days 1, 3, 5, and 7 of Differentiation

*Pax7 *was significantly upregulated in control young myoblasts on Days 5 and 7 of differentiation (Figure 2(j)) while* MyoD1 *in control young myoblasts was significantly upregulated on Day 5 of differentiation as compared to Day 0 (p<0.05) (Figure 2(k)). The expression of* Myf5 *was decreased in control young myoblasts on Days 1, 3, 5, and 7 of differentiation as compared to Day 0 (Figure 2(l)) while* MEF2C *in control young myoblasts was upregulated on Day 7 of differentiation (p<0.05) (Figure 2(m)). No significant change was observed on the expression of* IGF1R *(Figure 2(n)) and* MYH2 *(Figure 2(r)) on Day 1 till Day 7 of differentiation in control young myoblasts compared to Day 0 of differentiation. The expression of* MYOG *(Figure 2(o)) and* PTEN *(Figure 2(q)) in control young myoblasts was significantly increased on Days 3, 5, and 7 of differentiation while* TNNT1* in control young myoblasts was significantly upregulated on Day 3 of differentiation as compared to Day 0 of differentiation (p<0.05) (Figure 2(p)).

In control senescent myoblasts, an upregulation of* Pax7 *(Figure 2(j))*, TNNT1 *(Figure 2(p)), and* MYH2 *(Figure 2(r)) was observed on Days 5 and 7 of differentiation while* MyoD1 *(Figure 2(k)) and* Myf5 *(Figure 2(l)) were upregulated on Days 1, 3, 5, and 7 of differentiation, and* MEF2C *(Figure 2(m)),* MYOG *(Figure 2(o)), and* PTEN *(Figure 2(q)) were upregulated on Days 3, 5, and 7 of differentiation compared to Day 0 (p<0.05). No significant change was observed in the expression of* IGF1R* in control senescent myoblasts on Day 1 till Day 7 of differentiation compared to Day 0 (Figure 2(n)).

Treatment with 10 *μ*g/ml and 100 *μ*g/ml* C. vulgaris *was found to upregulated* Pax7 *in young myoblasts on Days 3, 5, and 7 of differentiation and in senescent myoblasts on Day 7 of differentiation (p<0.05) (Figure 2(j)); upregulated* MyoD1 *in both young and senescent myoblasts on Day 5 of differentiation (p<0.05) (Figure 2(k)); upregulated* Myf5 *in senescent myoblasts on Day 1 and 3 of differentiation (p<0.05), (Figure 2(l)); upregulated* MEF2C *in both young and senescent myoblasts on Day 5 of differentiation (p<0.05) (Figure 2(m)); upregulated* IGF1R *in both young and senescent myoblasts on Days 5 and 7 of differentiation (p<0.05) (Figure 2(n)); upregulated* MYOG *in young myoblasts on Days 3 and 5 of differentiation and in senescent myoblasts on Days 5 and 7 of differentiation (p<0.05) (Figure 2(o)); upregulated* TNNT1 *in young myoblasts on Days 3, 5, and 7 (p<0.05) (Figure 2(p)); upregulated* PTEN *in young myoblasts on Days 5 and 7 of differentiation and in senescent myoblasts on Days 1, 3, 5, and 7 of differentiation (p<0.05) (Figure 2(q)); downregulated* MYH2 *in young myoblasts on Day 1 of differentiation and upregulated* MYH2 *in young myoblasts on Days 3 and 7 of differentiation as compared to control (p<0.05) (Figure 2(r)).

### 3.4. MyomiRs Expression on Day 0 of Differentiation

**Figure 3:**
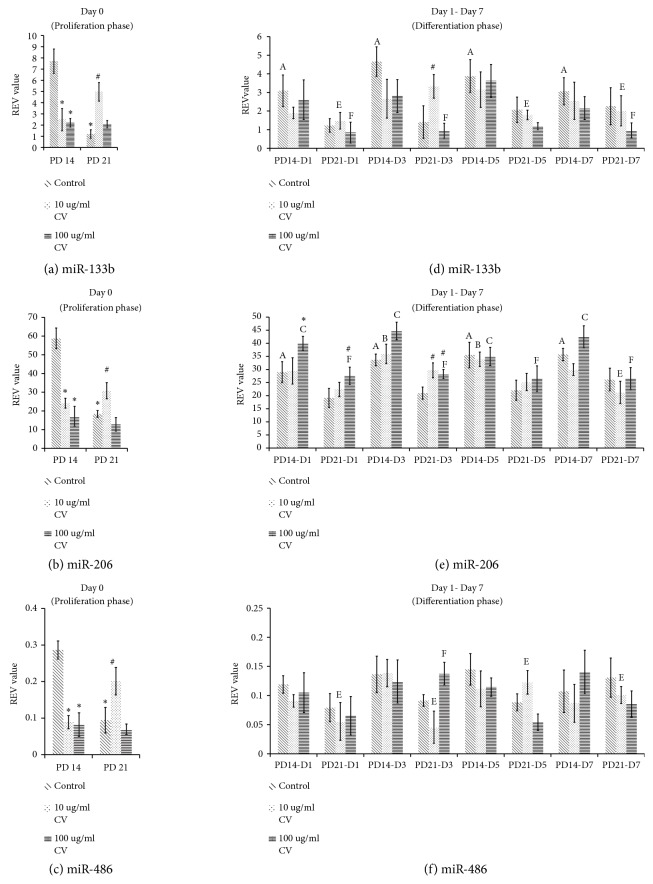
Effects of* C. vulgaris* treatment on the myomiRs expression at Day 0 of differentiation (a-c) and Days 1, 3, 5, and 7 of differentiation (d-f). The expression of myomiRs was determined in control and* C. vulgaris-*treated young and senescent myoblasts. The data are presented as the means ± SD, n = 3. *∗*p<0.05 significantly different compared to control young on respective day, ^#^p<0.05 significantly different compared to control senescent on respective day, ^A^p<0.05 significantly different compared to control young on Day 0, ^B^p<0.05 significantly different compared to young treated with 10 *μ*g/ml* C. vulgaris *on Day 0, ^C^p<0.05 significantly different compared to young treated with 100 *μ*g/ml* C. vulgaris *on Day 0, ^D^p<0.05 significantly different compared to control senescent on Day 0, ^E^p<0.05 significantly different compared to senescent treated with 10 *μ*g/ml* C. vulgaris *on Day 0, and ^F^p<0.05 significantly different compared to senescent treated with 100 *μ*g/ml* C. vulgaris *on Day 0, with a post hoc Tukey HSD test.

The expression of miR-133b, miR-206, and miR-486 in control senescent myoblasts was significantly decreased as compared to control young myoblasts (p<0.05) (Figures 3(a)–3(c)). A similar decrease in the expression of miR-133b, miR-206, and miR-486 in young myoblasts was observed with* C. vulgaris *treatment at 10 *μ*g/ml and 100 *μ*g/ml compared to control young (p<0.05). In senescent myoblasts however, treatment with 10 *μ*g/ml* C. vulgaris *was found to increase the expression of miR-133b, miR-206, and miR-486 compared to control senescent (p<0.05) (Figures 3(a)–3(c)).

### 3.5. MyomiRs Expression on Days 1, 3, 5, and 7 of Differentiation

In control young myoblasts, the expression of miR-133b was significantly decreased on Days 1, 3, 5, and 7 of differentiation compared to Day 0 (p<0.05) (Figure 3(d)). However, no significant change was observed in the expression of miR-133b in control senescent myoblasts on Day 1 till Day 7 of differentiation. A similar reduction in the expression of miR-206 in control young myoblasts was observed on Days 1, 3, 5, and 7 of differentiation as compared to Day 0 (p<0.05) (Figure 3(e)) with no significant change being observed in control senescent myoblasts. The expression of miR-486 was not significantly changed in both control young and control senescent myoblasts (Figure 3(f)). Treatment with 10 *μ*g/ml* C. vulgaris *significantly increased the expression of miR-133b and miR-206 in senescent myoblasts on Day 3 of differentiation compared to control (p<0.05) (Figures 3(d)-3(e)). Expression of miR-206 in young and senescent myoblasts was increased on Day 1 of differentiation with 100 *μ*g/ml* C. vulgaris *treatment compared to control (p<0.05) (Figure 3(e)). 100 *μ*g/ml* C. vulgaris *treatment also increased mir-206 expression in senescent myoblasts on Day 3 of differentiation compared to control (p<0.05). No significant change was observed in the expression of miR-486 with* C. vulgaris *treatment in young and senescent myoblasts (Figure 3(f)).

## 4. Discussion

Several mechanisms may contribute to sarcopenia and decrease the ability to reverse muscle atrophy in ageing. This includes decreased protein synthesis, reduction in neural function, hormonal deficit, chronic inflammation, oxidative stress, loss of mitochondrial function, inappropriate signaling in muscle due to inadequate nutrition, nuclear apoptosis, and reduction in satellite cell function [36]. In aged and sarcopenic muscles, satellite cell proliferation and differentiation may be diminished and consequently cause the decline in the regenerative potential. Modification in satellite cell function may affect differentiation and fusion of myoblast cells. It has been reported that, in senescent satellite cells, the level of MyoD protein and its DNA-binding activity were significantly reduced and delayed compared to young cells [33, 36] indicating the effect of ageing on myogenic regulatory factors (MFRs). Thus, elucidation of the mechanism involved is vital so that targeted intervention can be proposed.

The MRFs genes such as myogenic differentiation 1 protein (*MyoD1*), myogenic factor 5 (*Myf5*), myogenin (*MYOG*), and muscle-specific regulatory factor 4 (*Mrf4*) are myogenic specific which act for the activation of satellite cells [36]. The paired box protein (PAX7) controls the regulation of MYF5 and MYOD1 protein expression upon activation of quiescent satellite cells. The results of this study showed that the expression of* IGF1R, MEF2C, Myf5, MyoD1, MYOG, Pax7, TNNT1,* and* PTEN* was significantly decreased in control senescent myoblast cells as compared to control young myoblast cells on Day 0 of differentiation indicating downregulation of these genes during replicative senescence or cellular senescence of myoblast cells. The decrease in regenerative potential of aged muscles was reported to be associated with the reduction in satellite cell function and declined* Pax7* pool of myogenic stem cells [36]. Another study reported that* Pax3, Pax7, Myf5, MyoD, *and* MYOG *were significantly downregulated in the myoblasts extracted from elderly subjects compared to young subjects [37]. However, the* C. vulgaris*-treated senescent myoblast cells demonstrated a significantly increased expression of* IGF1R, MEF2C, Myf5, MYOG, Pax7,* and* PTEN* on Day 0 of differentiation as compared to control senescent myoblast cells indicating its role in the regulation of MRFs expression.

Our previous study showed that* C. vulgaris* improves the regenerative capacity of young and senescent myoblasts and promotes myoblast differentiation. Treatment with* C. vulgaris *resulted in decreased percentage of senescent myoblast cells stained positive for SA-*β*-gal. Additionally,* C. vulgaris* treatment improved the formation of myotubes in senescent myoblast cells as branched and multinucleated myotubes were observed. Fusion index and maturation index were also significantly higher on Day 5 of differentiation for* C. vulgaris*-treated young myoblast cells and Day 7 of differentiation for* C. vulgaris*-treated senescent myoblast cells as compared to control myoblasts. The percentage of young and senescent myoblast cells positively stained for myogenin was also observed to be significantly increased on Day 3 of differentiation compared to control myoblasts [34]. Thus, the promotion of myogenic differentiation by* C. vulgaris* was indicated and its potential in promoting muscle regeneration was further proven in the gene expression observed in this study. However, no significant difference was observed on MYH2 gene expression on Day 0 of differentiation in control and* C. vulgaris*-treated myoblasts for both young and senescent myoblasts. This observation may indicate that this gene is not affected in senescence and its expression is not regulated by* C. vulgaris. *

Besides MRFs and their regulators, microRNAs have been demonstrated to be involved in myogenic differentiation by modulating the expression of MRFs family in myogenesis. The MRFs, such as* MyoD, Myf5,* and* MYOG,* will activate the expression of a collection of myogenic microRNAs. This includes miR-1, miR-133, and miR-206, which are also known as myomiRs [37, 38]. The findings of this study showed that the expression of miR-133b, miR-206, and miR-486 was significantly decreased in control senescent myoblast cells compared to control young myoblast cells on Day 0 of differentiation indicating the involvement of these microRNAs in cellular senescence of myoblasts. However, a significant increase was observed in* C. vulgaris*-treated senescent myoblast cells on Day 0 of differentiation which may results in the promotion of myoblasts proliferation. A previous study reported that myoblast extracted from elderly subjects showed downregulation of miR-133b [37].

After limited rounds of proliferation, the majority of satellite cells enter the myogenic differentiation program and begin to fuse with each other to form new myofibers [38]. The paired box 7 (*Pax7*) is one of the earliest markers during myogenesis. It has been reported that* Pax7* regulates muscle marker genes such as* Myf5* and* MyoD* towards differentiation [39]. Proliferating myoblasts continue to express* Pax7*. However, in contrast to their quiescent progenitors, it also expresses* MyoD.* A reduction in* Pax7* along with the induction of muscle-specific transcription factor* MYOG* marks myoblast that have entered the differentiation phase and initiate cell cycle withdrawal [7]. The* Pax7 *was also reported to play a dual role in myogenesis regulation by activating commitment to the myogenic program and simultaneously prevent terminal differentiation [40]. Upon commitment to terminal differentiation, upregulation of* MYOG* will directly or indirectly downregulate* Pax7*. A high ratio of* Pax7* to* MyoD* was observed in quiescent satellite cells to maintain satellite cells in their quiescent state. An intermediate ratio of* Pax7* and* MyoD* will allow the satellite cells to proliferate, but not differentiate. However, satellite cells with a low* Pax7* to* MyoD* ratio will begin to differentiate and a further reduction in* Pax7* level was observed with activation of* MYOG *[38]. In this study, a similar finding on* Pax7 *and* MyoD* ratio was observed. The expression of* Pax7 *was increased in early days of differentiation but later decreased towards the end of differentiation for both young and senescent myoblasts while the expression of* MyoD* and* MYOG* was increased. However, with* C. vulgaris* treatment, the expression of* Pax7 *was increased compared to its untreated control on each day of differentiation, in both young and senescent myoblasts indicating the promotion of proliferation and differentiation of myoblast by the alga.

Coinciding with or occurring soon after the upregulation of* MYOG*, differentiating myoblasts will initiate the expression of various genes encoding for structural proteins, such as sarcomeric myosin which fuse into myotubes [7]. Proliferation of satellite cells leads to the formation of new stem cells, which is maintained in undifferentiated state and myogenic precursor cells that express MRFs for muscle differentiation, which includes* MyoD, Myf5, *and* MYOG. *The conversion from quiescent state to the activated state is rapidly followed by muscle differentiation, with myosin heavy chains (*MyHCs*) expression and myoblast fuse with each other for the formation of myotubes. The activated satellite cells will begin to express either* MyoD* or* Myf5. *However, most cells will express both* Myf5 *and* MyoD* simultaneously, in which* MYOG* will be expressed by the cells followed by the expression of both* MyoD *and* Myf5. *Many cells will ultimately express all MRFs simultaneously. The activation of satellite cells followed by proliferation and fusion will occur after one day of injury and up to seven days [41]. In another study, it was described that* MyoD* and* Myf5* were quickly upregulated in young myoblasts during the first hours of differentiation, followed by the expression of cell cycle regulator p57 and* MYOG* [33]. However, in senescent myoblast, both delay expression and downregulation of* MyoD* were observed resulting in failure of* Myf5* activation. The delayed expression of p57 and* MYOG *was also observed in senescent myoblast.

The results of our study showed that the expression of* MyoD, Myf5, *and* MYOG* was increased in both young and senescent myoblasts with the increasing number of differentiation days. The expression of these transcription factors was also significantly increased with the treatment of* C. vulgaris*, in both young and senescent myoblasts, thus confirming its potential in promoting myoblast differentiation. A previous study reported that myoblast coexpressing both* Myf5 *and* MyoD* exhibits the intermediate growth and differentiation propensities, with the expression of* MyoD* peaks in mid G_1_ and* Myf5* expression is maximal at the G_0_ and G_2_ phases of the cell cycle [38].

A previous study reported that serum response factor (SRF) positively regulates the expression of* MyoD *in proliferating myoblasts by binding to serum response element (SRE) within the* MyoD *regulatory region. However, SRF only drives low levels of MyoD expression due to its activity which is hindered by cyclin D1 induced dependent kinase 4 (Cdk4). Induction of myocyte enhancer factor-2 (*MEF2*) expression prior to differentiation enables* MEF2 *to outcompete SRF for the SRE binding site and consequently resulted in high levels of* MyoD* expression and initiation of differentiation [38]. The* MEF2 *family of human transcription factors consists of four proteins,* MEF2A, MEF2B, MEF2C, *and* MEF2D, *in which the expression of* MEF2C *was determined in this study. Our finding showed that the expression of* MEF2C* was increased in both young and senescent myoblasts throughout the differentiation day. Treatment with* C. vulgaris* was found to significantly increase the expression of* MEF2C* as compared to its control on respective day of differentiation.* MEF2C *is involved in the regulation of cytoskeletal structures and loss of* MEF2C* in skeletal muscle will result in improper sarcomere organization. Its isoform,* MEF2Cα2, *is predominantly expressed in skeletal muscle and promotes muscle-specific gene expression and myogenic differentiation [42, 43]. Thus upregulation of* MEF2C *observed in this study may indicate promotion of myogenic differentiation by* C. vulgaris *throughout the differentiation day.

Insulin-like growth factors (*IGFs*) exist as two isoforms,* IGF-I *and* IGF-II *that play vital roles in the regulation of satellite cell activity. The* IGF *exerts pleiotropic functions, such as anti-inflammation, cell migration, and stimulation of both proliferation and differentiation in satellite cells, which is mediated by the binding of* IGF-I *to* IGF-I *receptor (*IGF1R*), a ligand-activated receptor tyrosine kinase. The expression level of* IGF1R* is critical for the regulation of muscle development due to its function in directly regulating the intracellular responsiveness of muscle cell to the extracellular* IGF* signal. The activation of* IGF1R* in satellites cells will promote the expression of MRFs, such as* MYOG*. The* IGF1R* activates two primary signaling pathways: (1) upon ligand binding, IGF1R becomes auto phosphorylated and induces the phosphatidylinositol 3-kinase (PI3K)/Akt pathway which is involved in myoblast differentiation and (2) IGF1R activates the Ras/Raf/extracellular response kinases (ERKs) cascade, resulting in the activation of other protein kinases and transcription factors for satellite cell proliferation [38, 44]. The results of this study showed that the expression of* IGF1R *was maintained throughout the differentiation in both young and senescent myoblast cells. However, with* C. vulgaris* treatment, the expression of* IGF1R* was significantly increased in young and senescent myoblasts suggesting promotion of myoblast differentiation by* C. vulgaris*.

Troponin T (*TnT*) is a central player in the calcium regulation of actin thin filament function and is essential for the contraction and relaxation of striated muscles.* TnT* exists as three isoforms,* TNNT1 *and* TNNT3 *which are specifically expressed in slow and fast twitch skeletal muscle fibers and* TNNT2 *which is expressed specifically in cardiac muscle [45]. The deletion of phosphatase and tensin homologue (*PTEN*) in quiescent satellite cells will cause spontaneous activation of satellite cells and undergo premature differentiation without proliferation, resulting in satellite cells depletion and defective regenerative function of skeletal muscle in response to injury.* PTEN* is vital for satellite cell homeostasis and the deletion of* PTEN *in embryonic myogenic progenitors resulted in defective and depleted satellite cells and consequently causes failure to myoblast regeneration [46, 47].

The myofibers consist of repeated actin and myosin myofibrils forming sarcomere, which is the basic functional unit of skeletal muscle which is involved in muscle contraction. Skeletal muscle fibers can be categorized into a slow-contracting/fatigue-resistant type and fast-contracting/fatigue-susceptible type muscle fibers. It differs in terms of their myosin heavy chains (*MyHC*) isoforms and types of metabolism [38]. Various forms of* MyHCs *are encoded by a large family of sarcomeric* MYH *genes expressed in striated muscles. The* MYH2* gene produces MyHC-2A protein with fast type 2A fibers [48]. In this study, the expression of* TNNT, PTEN, *and* MYH2 *was significantly increased in both young and senescent myoblast cells during differentiation. The expressions of* TNNT1, PTEN, *and* MYH2* were significantly increased with* C. vulgaris* treatment indicating its potential in the promotion of muscle differentiation and regeneration.

The microRNAs play an important role during muscle proliferation and differentiation by regulating the expression of a number of transcription factors and signaling molecules required for myogenesis [49]. miR-133b and miR-206 are located at human chromosome 6p12.2 with tissue specificity towards skeletal muscle, while mir-486 is located at human chromosome 8p11.21 with tissue specificity towards heart and skeletal muscle [16]. The function of each myomiR is summarized as follows: miR-133b promotes myoblasts differentiation and fusion, regeneration, alternative splicing regulation, chromatin remodeling, cell fate regulation, and proapoptotic; miR-206 promotes myoblast differentiation and regeneration, regeneration of neuromuscular synapses, chromatin remodeling, antiangiogenic, proapoptotic, oxidative stress control, and antimigration; and miR-486 promotes myoblast differentiation and fusion, alternative splicing regulation, antiapoptotic, and promigration [50]. Both miR-133b and miR-206 are required for skeletal muscle differentiation.

Generally, the cell signaling pathways targeted by miR-206 tend to have opposing functions to the regulatory pathways targeted by miR-133b, in which miR-206 acts to promote myogenic differentiation and miR-133b maintains the undifferentiated state and promotes cell growth; thus coexpression of myomiRs will help in the maintenance of homeostasis under normal cellular conditions. Since the downregulation of myostatin permits expression of miR-133b/-206 and* MyoD,* and* MYOG* binds to miR-206 promoter, it was suggested that miR-133b/-206 expression in the muscle may also be partly controlled by* MyoD* and* MYOG *[51]. In this study, the expression of miR-133b in young myoblasts was significantly increased during differentiation as compared to Day 0. A similar increase in miR-133b was not observed in senescent myoblasts during differentiation suggesting slower regenerative capacity in ageing. Treatment with* C. vulgaris* however was found to increase the expression of miR-133b in senescent myoblasts on Day 3 of differentiation.* C. vulgaris *treatment also significantly increased the expression of miR-206 in young myoblasts on Day 1 and Day 3 of differentiation while the expression of miR-206 in senescent myoblasts was increased only on Day 3 of differentiation. These findings may indicate the potential of* C. vulgaris *in promoting myogenic differentiation. A previous study reported that increased miR-206 expression in proliferating myoblasts was initiated by the proximal (PROX) promoter. miR-206 is located within the intron of linc-MD1 and is transcribed autonomously under the control of its own PROX promoter [51]. This microRNA is upregulated by* MyoD* and targets* Pax3 *and* Pax7* mRNA for myogenic differentiation regulation. Thus, through this miR-206 mediated negative feedback mechanism,* MyoD* facilitates progression of myoblasts towards terminal differentiation [52, 53].

**Figure 4:**
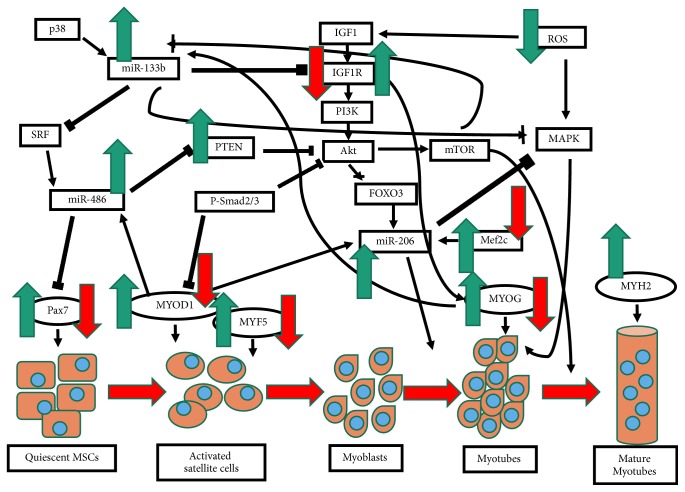
The regulation of genes and myomiRs expression in the IGF/PI3K/Akt signaling pathway during myogenesis. Control senescent myoblast cells demonstrated a significantly decreased expression of genes on Day 0 of differentiation, as indicated by red arrow. However, the expression of genes and myomiRs was significantly increased in* C. vulgaris-*treated young and senescent myoblasts cells throughout the differentiation day, as indicated by green arrows.

Figure 4 summarizes the myogenesis process which is controlled by a group of myogenic regulatory factors (MRFs) that command the progression from quiescence to activation, proliferation, and differentiation of muscle satellite cells (MSCs). This process will lead to the transformation of individual satellite cell into a syncytial contractile myofibers [38]. Quiescent muscle satellite cells (MSCs) are characterized by the expression of transcription factor* Pax7*. These MSCs will exit from the quiescent state and will be activated in the presence of stimuli such as damaged to the environment of satellite cells [38, 54]. The proliferating MSCs will later express* MyoD1 *and Myf5 for its activation and promotion of entry into the cell cycle, during muscle fiber development and muscle regeneration [54]. It will be further promoted to differentiate by inducing* MyoD1 *expression. The* MYOG *expression will initiate terminal differentiation and fusion in immature myotubes and the expression of myosin heavy chain (*MYH2*) in mature myotubes will activate muscle-specific structural and contractile genes, thereby impacting muscle function [38, 54].* Pax7 *will be downregulated upon the activation of myogenic regulatory genes such as* Myf5 *and* MyoD1 *during the initiation of myogenesis [55]. Thus, the activated satellite cells will return to quiescence state. This is important to maintain the satellite cell pool for long term muscle integrity [38].

The phosphatidylinositol-3-kinases (PI3K)/Akt signaling pathway is the major signaling pathway which regulates muscle protein synthesis by modulating IGF-1 and insulin expression [4, 56] that promote protein synthesis and muscle hypertrophy by interacting with their respective tyrosine kinase receptors to phosphorylate the insulin receptor substrate (IRS-1). PI3K/Akt will then activate and stimulate mTOR to further promote protein synthesis [4] (Figure 4). The presence of low levels of reactive oxygen species (ROS) plays an important role in inducing the upregulation of growth factors such as IGF-1 [57].

The IGF-1 targets miR-206 and miR-133 while the PI3K/Akt pathway inhibits FoxO3 expression for the upregulation of miR-206 which resulted in the promotion of myoblast differentiation [50]. The miR-133 has a conserved and functional binding site in the 3′′-UTR of IGF-1R, which consequently declined in IGF-1R abundance [14]. The miR-133 will promote myoblast proliferation by targeting the mRNA for serum response factor (SRF) for miR-486 activation [54]. The miR-486 will be highly upregulated during muscle differentiation by targeting* Pax7 *and consequently accelerated myoblast differentiation [58]. While the miR-133b will suppress myoblast proliferation and promote myoblast differentiation via mitogen-activated protein kinase (MAPK) regulation, by downregulating its transducers, which includes fibroblast growth factor receptor 1 (FGFR1) and protein phosphatase 2A catalytic subunit (PP2AC) [59]. The MAPK is also responsible in ensuring sufficient myoblast accumulation for fusion and myotubes formation. Thus, MAPK negatively regulates miR-133b expression and therefore maintaining proliferation until differentiation induction by a significant upregulation of miR-133b occurs [50].

The expression of myomiRs is dependent on the expression of myogenic regulatory factors such as* MyoD1*,* MYOG, *and* Mef2c *which regulate the balance between myoblast proliferation and differentiation [55]. The* Mef2c *is also responsible for the promotion of miR-206 [50]. The inhibition of* Pax7 *by miR-486 will result in* MyoD1 *upregulation and further enhancement of its own expression since miR-486 is directly regulated by* MyoD1 *and SRF [58]. The PTEN is downregulated by miR-486, thereby increasing pAkt, which in turn phosphorylates FoxO resulting in its activation. A previous study reported that muscle wasting is limited by the inactivation of FoxO [14]. The downregulation of miR-486 in normal myoblasts also leads to impaired migration and myoblast fusion [50].

## 5. Conclusion

*C. vulgaris *may promote myogenic differentiation as indicated by the upregulation of MRFs and myomiRs expression in culture. The modulatory effects of* C. vulgaris *on the expression of MRFs and myomiRs may indicate its potential in promoting muscle regeneration and ameliorating sarcopenia.

## Figures and Tables

**Table 1:** Sequence of primers used in gene expression analysis.

Gene	Forward primer	Reverse primer
*GAPDH*	5′-TCCCTGAGCTGAACGGGAAG-3′	5′-GGAGGAGTGGGTGTCGCTGT-3′
*Pax7*	5′-GTGCCCTCAGTGAGTTCGAT-3′	5′-GTTCCGACTCCACATCCGAG-3′
*Myf5*	5′-TCACCTCCTCAGAGCAACCT-3′	5′-ATTAGGCCCTCCTGGAAGAA-3′
*MyoD1*	5′-AGGGGCTAGGTTCAGCTTTC-3′	5′-GCTCTGGCAAAGCAACTCTT-3′
*MYOG*	5′-CAGTGCCATCCAGTACATCG-3′	5′-AGGTTGTGGGCATCTGTAGG-3′
*PTEN*	5′-ACTTGAAGGCGTATACAGGAC	5′-AATGTCTTTCAGCACAAAGAT
	CA-3′	TGTA-3′
*IGF1R*	5′-TGGAGTGCTGTATGCCTCTG-3′	5′-CCCTTGGCAACTCCTTCATA-3′
*MEF2C*	5′-GGGGACTATGGGGAGAAAAA-3′	5′-ACAGCTTGTTGGTGCTGTTG-3′
*MYH2*	5′-CAAACATGAGAGGCGAGTGA-3′	5′-CTGGAGCTTGCGGAATTTAG-3′
*TNNT1*	5′-TGGAGCTGCAGACACTCATC-3′	5′-CTTGGCCTCTTCCTCTTCCT-3′

**Table 2:** The targeted sequence used in myomiRs expression analysis.

miRNA primer	Assay ID	Target sequence
hsa-miR-133b	480871_mir	5′-UUUGGUCCCCUUCAACCAGCUA-3′
hsa-miR-206	477968_mir	5′-UGGAAUGUAAGGAAGUGUGUGG-3′
hsa-miR486-5p	478128_mir	5′-UCCUGUACUGAGCUGCCCCGAG-3′
hsa-miR-191-5p	477952_mir	5′-CAACGGAAUCCCAAAAGCAGCUG-3′

## Data Availability

The data used to support the findings of this study are included within the article.
